# The Lords' Committee

**Published:** 1891-03-21

**Authors:** 


### The Lords' Committee.

The Brompton Consumption Hospital was the institution of which the management was inquired into by the Lords' Committee on March 12th. Mr. Henry Dobbin, examined by the Chairman, said he had been thirty-three years Secretary of the Brompton Consumption Hospital. All chest diseases were also treated at the hospital. It was founded in 1841, and was generally looked upon as suitable for the purposes for which it was intended. There were two buildings—one on each side of the road—and there were 184 beds in the old, and 137 in the new building. When full there were about 300 beds occupied daily. There were no paying patients. The patients were nursed by the hospital staff, at the head of which was the Lady Superintendent, who resided in the hospital, and had a salary of £150 a-year and everything found. She also received twenty guineas as a fee for lecturing to the nurses. There were seven Sisters—one at the head of each gallery for day duty; two night Superintendents—one for each building. There were sixty-one regular staff nurses and probationers, and nine extra nurses. The day nurses worked from 7 a.m. to 9 p.m., and were off three times a week from six to eight, once a month from two to ten, and once a month from twelve to ten, and on one Sunday a month from twelve to nine. They had an annual holiday of sixteen days. There were ward maids to do the heavy and menial work. On being questioned as to the drainage of the hospital, Mr. Dobbin said that two years ago the whole of the drainage of the old building was overhauled and entirely reconstructed. The water supply was from the Chelsea Waterworks Company, and was, practically, constant. There were most complete fire arrangements at the hospital, with hydrants from the top to the bottom, and there was a regular drill under the superintendence of the local fire inspector. Although there was no professional audit of the accounts, they were kept according to the system provided by the rules, and were audited by business men. Dr. Theodore Williams, senior physician of the Brompton Hospital, next examined, stated that he would frankly admit, while the position of the hospital was, at the time it was built, considered the best in London, that the best places for consumption hospitals was not in towns; they ought to be in the pure air. Still, at Brompton, every measure was taken for the benefit of the patients, to secure as much air as possible, and at an even temperature. The system of ventilation was of the very best. A proof of the need of consumption hospitals was given in the large number established throughout the country since that at Brompton was started. Anxious inquiries also came from the Continent and America. Medical men had come from Paris, Berlin, St. Petersburg, and America to study, with the view of imitating, the arrangements at Brompton. He would not go so far as to say that the majority of cases there were cured, but they were greatly improved. Mr. Taylor, M.R.C.P., attached to the West London Hospital, spoke in condemnation of the out-patient departments of hospitals as being greatly abused by persons who came with but slight ailments, and who could also afford to pay something, and were not, therefore, fit subjects for gratuitous hospital treatment. Mr. A. C. P. Coote, in examination by the Chairman, said that for three years he had been Secretary to the Lock Hospital, which had a female branch and a rescue home in the Harrow Road, and a male department in Dean Street, Soho. The hospital was managed economically, the cost per bed being between £40 and £45 a-year. Their subscriptions last year amounted to about £1,500; their donations were £2,100; legacies, £1,000; payments from unions, £1,400. A dinner which was held brought in about £2,000. Their gross receipts were between £7,000 and £8,000, which gave them enough to meet their expenditure for the year; and they had been able to reduce to £4,000 their debt, which three years ago was £6,000. They owed £1,750 to their bankers, and the rest to tradesmen. He might mention as an interesting fact that they had received voluntary contributions from out-patients averaging a shilling apiece all round, and which had last year amounted to a total of £1,163.

On Monday last, Mr. P. Michelli, Secretary of the Seamen's Hospital Society, Greenwich, was examined. He stated that he had been Secretary to the institution for four years. The hospital, which was formerly the old Dreadnought Hospital, was entirely free, and was specially intended for seamen, although urgent cases of landsmen, women, and children were taken in. There were 225 beds at the hospital, and 14 at the branch. It would be a very good thing to have a central body to audit the accounts. That body ought not, however, to have any control over the hospitals except that of inspection. It would be useful in suggesting, where it was desirable, that new hospitals should be erected. He rather favoured the suggestion of Sir Henry Longley as to the formation of a central body.

